# Supplementary material for: Improvements in airflow characteristics and effect on the NOSE score after septoturbinoplasty: A computational fluid dynamics analysis
Source: PLoS One. 2022 Nov 17;17(11):e0277712. doi: 10.1371/journal.pone.0277712 (PMC9671303; doi:10.1371/journal.pone.0277712)
Supplement: Attachment — Submitted filename: response to reviewer_2nd_rebuttal_v2.docx [file pone.0277712.s002.docx]

Rebuttal letter

Manuscript Number: PONE-D-22-14744R1

Dear Editor-in-Chief of *PLOS ONE*,

We have revised our manuscript entitled “Improvements in airflow characteristics and effect on the NOSE score after septoturbinoplasty: A computational fluid dynamics analysis.”

In the revised paper, we have tried to address the comments raised by the reviewer and

the editor. We found those comments helpful once again. We hope that we satisfyingly addressed those issues and that our revised manuscript will be suited for publication.

Sincerely,

**Corresponding author:** Yong Gi Jung, MD, PhD

Address: Department of Otorhinolaryngology, Head and Neck Surgery, Samsung Seoul Hospital, 81 Irwon-Ro Gangnam-gu. Seoul, Korea, 06351

Tel.: (82) 02-3410-3579

Fax.: (82) 02-3410-3879

E-mail: Ent.jyg@gmail.com

**Reply to the Academic editor**

1. Have the authors made all data underlying the findings in their manuscript fully available?

The [PLOS Data policy](http://www.plosone.org/static/policies.action#sharing) requires authors to make all data underlying the findings described in their manuscript fully available without restriction, with rare exception (please refer to the Data Availability Statement in the manuscript PDF file). The data should be provided as part of the manuscript or its supporting information or deposited to a public repository. For example, in addition to summary statistics, the data points behind means, medians and variance measures should be available. If there are restrictions on publicly sharing data—e.g., participant privacy or use of data from a third party—those must be specified.

(*Response*)

To support author compliance of the PLOS data policy, we have deposited our data to the public repository of Dryad (which is recommended by PLOS One). Our data can be accessed by the following link: <https://orcid.org/0000-0001-5729-6912>. In this repository, we have deposited the following 4 files (one readme file and three datafile). The readme file (Microsoft Word format) summarizes the title, authors, abstract and the data files. Three datafiles (Microsoft Excel format) contain the raw data used in the present study.

(1) README File
(2) flow_partition_nasal_resistance_data_for_Dryad

- Flow partition, unilateral nasal resistance, bilateral nasal resistance of the pre- and postoperative models

(3) relative_humiduty_air_temperature_data_for_Dryad

- Relative humidity, air temperature of the pre- and postoperative models

(4) surface_heat_flux_data_for_Drayad

- Surface heat flux (sensible and latent heat) in the MOS and LOS of the Area-1 of the pre- and postoperative models
- Surface heat flux (sensible and latent heat) in the MOS and LOS of the Area-2 of the pre- and postoperative models

We have indicated the above data deposition in the first page of the revised paper as follows:

“Data availability: Data for this study are available in DRYAD at <https://doi.org/10.5061/dryad.nk98sf7wj> “

**Reply to Reviewer #1**

The authors appreciate this reviewer’s comment once again. We have tried to address the reviewer’s comment more clearly to the best of our knowledge in this report and the revised manuscript.

**Responses to specific comments:**

1. Reviewer #1: The indication of septoplasty and turbinoplasty were not clearly stated in all patients. Do all patients need both procedures? A control arm of septoplasty alone and turbinoplasty alone should be done to compare the improvement of combined procedures.

(*Response*)

Thank you very much for your thoughtful comments.

The following sentence has been added to the methods section so that readers can more clearly understand the patient group selection in this study.

**“Both septoplasty and turbinoplasty were recommended to secure the patient's nasal airway as much as possible, and septoturbinoplasty was performed in all subjects after obtaining consent.”**

And the research ideas suggested by the reviewers were added to the discussion section as in the following sentence and marked with highlighted.

**“This study was conducted on patients who underwent septoplasty and turbinoplasty together. Still, there is a plan to research to elucidate the proper indications for each procedure in the future.”**

The purpose of this study was not to compare the pattern of CFD change before and after surgery in septoplasty and turbinoplasty, but to compare symptom improvement and CFD parameters of patients who underwent both procedures. Therefore, please understand the purpose of this study.

However, the reviewer's opinions are clinically critical, and I think it would be meaningful to separate and compare septoplasty and turbinoplasty. Because septoturbinoplasty is performed in almost all patients at our institution, it is difficult to conduct a retrospective study on the above topic, but a prospective study can be possible.

Thank you for giving me a good idea, and I will plan further research on the above topic. So please stay tuned for further study by our team.

Appreciate your helpful comment.
